# Predicted *STAT1*-*BRAF* and *IRF7*-*MET* Regulatory Links Are Associated with MAPK Pathway Reactivation and Targeted Therapy Resistance in Melanoma

**DOI:** 10.3390/ijms27188031

**Published:** 2026-09-09

**Authors:** Hao Fu, Mengyao Wang, Haibo Zhu, Weihua Li, Xiaopei Shen, Haidan Yan, Jun He

**Affiliations:** 1 Department of Bioinformatics, Fujian Key Laboratory of Medical Bioinformatics, Institute of Precision Medicine, School of Medical Technology and Engineering, Fujian Medical University, Fuzhou 350122, China; haofu@fjmu.edu.cn (H.F.); wangmy2022@gmail.com (M.W.); bioinfo_zhb@163.com (H.Z.); lw2696250@gmail.com (W.L.); xshen@fjmu.edu.cn (X.S.); 2 Key Laboratory of Gastrointestinal Cancer (Fujian Medical University), Ministry of Education, Fuzhou 350122, China

**Keywords:** melanoma, *BRAF* inhibitor, acquired resistance, single-cell RNA sequencing, MAPK pathway reactivation, *STAT1*, *IRF7*, *MET*

## Abstract

Resistance to *BRAF* inhibitors (BRAFi), alone or with MEK inhibitors (MEKi), limits durable responses in *BRAF*-mutant melanoma. To characterize resistance-associated cell-state evolution, we analyzed 674 melanoma cells from six mice bearing tumors from a single patient-derived *BRAF* V600E-mutant melanoma xenograft (PDX) lineage before treatment, during initial regression, at minimal residual disease, and at resistant regrowth. Unsupervised clustering based on a *BRAF*-centered network feature set comprising 2506 candidate genes identified six transcriptional states, which were characterized using transcriptomic analyses. Cluster 4 was detected exclusively at resistant regrowth, a phase marked by MAPK pathway reactivation, and exhibited enhanced JAK-STAT/interferon signaling and increased *STAT1*, *STAT2*, *IRF7*, *IRF9*, and *RELB* regulon activities. Network inference predicted *STAT1*-*BRAF* and *IRF7*-*MET* regulatory links, suggesting candidate routes to MAPK reactivation through *BRAF* overexpression and *MET*-mediated bypass signaling. External analyses partially recapitulated the resistance-associated transcriptional program in independent melanoma cell-line datasets and yielded limited, inconclusive evidence for the predicted *STAT1*-*BRAF* association in public perturbation datasets. Cluster 2 represented a pre-existing proliferative state whose signature was associated with shorter progression-free survival in pretreatment clinical cohorts. Together, these findings distinguish a therapy-associated acquired-resistance state from a pre-existing proliferative resistance-associated state and nominate the predicted *STAT1*-*BRAF* and *IRF7*-*MET* links for functional validation.

## 1. Introduction

Melanoma is one of the most aggressive malignancies of the skin [1,2,3], accounting for approximately 332,000 new cases and 59,000 melanoma-related deaths worldwide in 2022 [4]. While localized melanoma is highly curable by surgical resection, advanced or metastatic disease is characterized by rapid progression and substantial mortality. The clinical management of advanced melanoma has been transformed by systemic interventions, particularly immune checkpoint blockade and molecularly targeted therapies [2]. However, the long-term efficacy of these treatments remains limited by intrinsic and acquired therapeutic resistance. Consequently, metastatic melanoma continues to carry a poor prognosis, with a five-year survival rate of approximately 30% [4].

Among the molecular alterations driving melanoma, the *BRAF* V600E mutation is one of the most prevalent, occurring in approximately 50% of cases [5]. This mutation constitutively activates the downstream RAF/MAPK signaling pathway and promotes tumor proliferation [6]. *BRAF* inhibitors (BRAFi), such as vemurafenib, are standard therapeutic agents [7]; however, their efficacy is limited, with a median progression-free survival (PFS) of approximately 9 months [8]. To improve therapeutic efficacy, BRAFi are commonly combined with MEK inhibitors (MEKi) [9]. This combination strategy increases the response rate from 54% to 76% [10] and extends median PFS and overall survival to approximately 12 and 24 months [11], respectively. Nevertheless, although most patients initially respond favorably, acquired resistance commonly develops after several months of treatment.

Resistance mechanisms are diverse and frequently involve reactivation of the MAPK pathway through increased receptor tyrosine kinase (RTK) activity, *BRAF* overexpression, or bypass signaling [12,13]. Alternative pathways, including PI3K/AKT signaling, as well as tumor microenvironment-derived factors, also contribute to therapeutic evasion [14,15]. Recent advances in single-cell RNA sequencing (scRNA-seq) have reshaped our understanding of melanoma progression by enabling the identification of distinct drug-tolerant and resistant cell states [16,17]. However, the dynamic transcriptional shifts that occur as melanoma cells progress from initial drug response to terminal resistance remain incompletely understood, largely because longitudinal patient-derived samples are rarely accessible and conventional in vitro models do not fully capture temporal evolution under active therapy [18]. Serially sampled patient-derived xenograft (PDX) models provide a valuable alternative for tracking these in vivo processes; however, the transcriptional regulatory programs linking treatment-associated cell-state changes to MAPK pathway reactivation remain incompletely defined.

To address these gaps, we analyzed longitudinal scRNA-seq data from a *BRAF* V600E-mutant melanoma PDX lineage sampled across four therapeutic phases (GSE116237). We aimed to characterize changes in melanoma cell states under targeted therapy and to identify candidate transcriptional regulators associated with resistance. Clustering based on a *BRAF*-centered network feature set was followed by full-transcriptome pathway, pseudotime, and regulon analyses to distinguish pre-existing from therapy-associated resistance states and identify candidate regulatory mechanisms linked to MAPK pathway reactivation.

## 2. Results

### 2.1. BRAF-Centered Network Analysis Delineates Melanoma Cell States During Targeted Therapy

**Figure 1:**
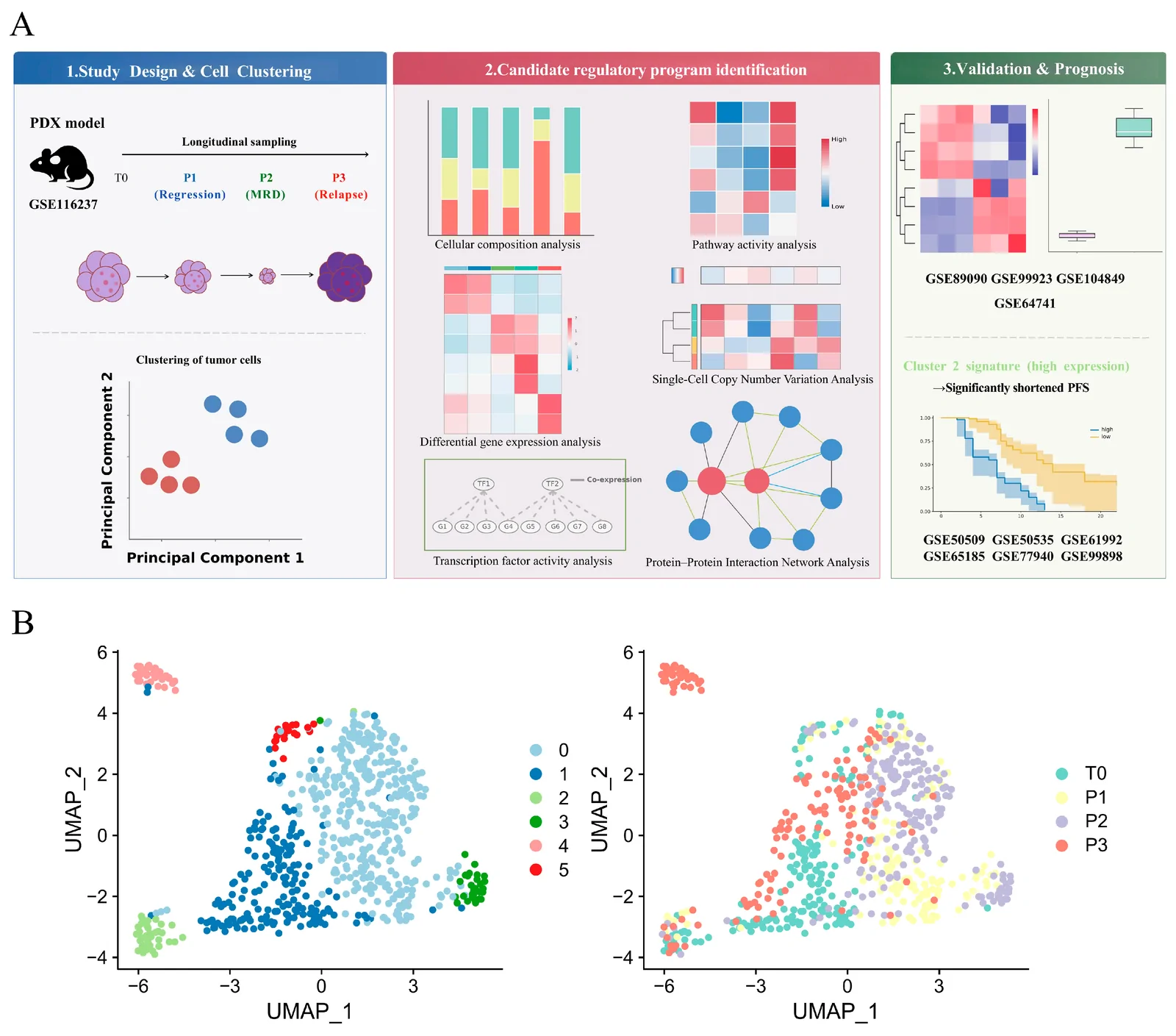
Study design and *BRAF*-centered delineation of melanoma cell states during targeted therapy. (**A**) Schematic overview of the analytical workflow, including longitudinal scRNA-seq analysis across four therapeutic phases of the MEL006 PDX model, *BRAF*-centered clustering and downstream characterization of resistance-associated transcriptional programs, and external evaluation in independent cell-line and clinical datasets. ((**B**), left) UMAP representation of 674 melanoma cells partitioned into six transcriptionally distinct clusters (Clusters 0–5) using the *BRAF*-centered network feature set. ((**B**), right) Distribution of cells across four therapeutic phases: untreated (T0), initial regression (P1), minimal residual disease (P2), and resistant regrowth (P3).

To characterize cellular heterogeneity under BRAF inhibition, unsupervised clustering of 674 melanoma cells from a longitudinal PDX dataset was performed using highly variable genes selected from a *BRAF*-centered network feature set (Figure 1A). The dataset encompassed four therapeutic phases: untreated (T0), initial regression (P1), minimal residual disease (P2), and resistant regrowth (P3). This focused clustering strategy was used to resolve transcriptional states potentially associated with MAPK signaling and therapeutic adaptation.

*BRAF*-centered clustering partitioned the melanoma cells into six transcriptionally distinct clusters (Figure 1B). Their distributions varied across therapeutic phases. Cells from the treatment-responsive P1 and P2 phases largely occupied adjacent regions of the UMAP space, whereas T0 and P3 cells showed partial convergence within another region of the embedding. These stage-associated distributions were consistent with dynamic transcriptional remodeling during targeted therapy and suggested that P3 included a transcriptional configuration distinct from the initial drug-responsive states. This organization motivated further characterization of cluster composition and functional programs associated with resistant regrowth.

Clustering robustness was evaluated using resolution sensitivity, Reactome pathway coverage, and whole-transcriptome comparison. The six-cluster structure remained stable across Louvain resolutions of 0.28–0.31, with additional subdivision beginning at 0.32 (Figure S1). The 2506-gene set covered all 34 genes in the Reactome RAF activation pathway and 209 of 265 genes (78.87%) in the broader RAF/MAP kinase cascade (Table S1). Whole-transcriptome clustering yielded four broader groups, but the projected Cluster 2 and Cluster 4 assignments remained spatially distinct (Figure S2). Thus, the focused feature set provided finer state resolution, whereas the separation of Clusters 2 and 4 was retained in the whole-transcriptome feature space.

### 2.2. Identification and Functional Characterization of a Candidate Acquired Resistance-Associated State

**Figure 2:**
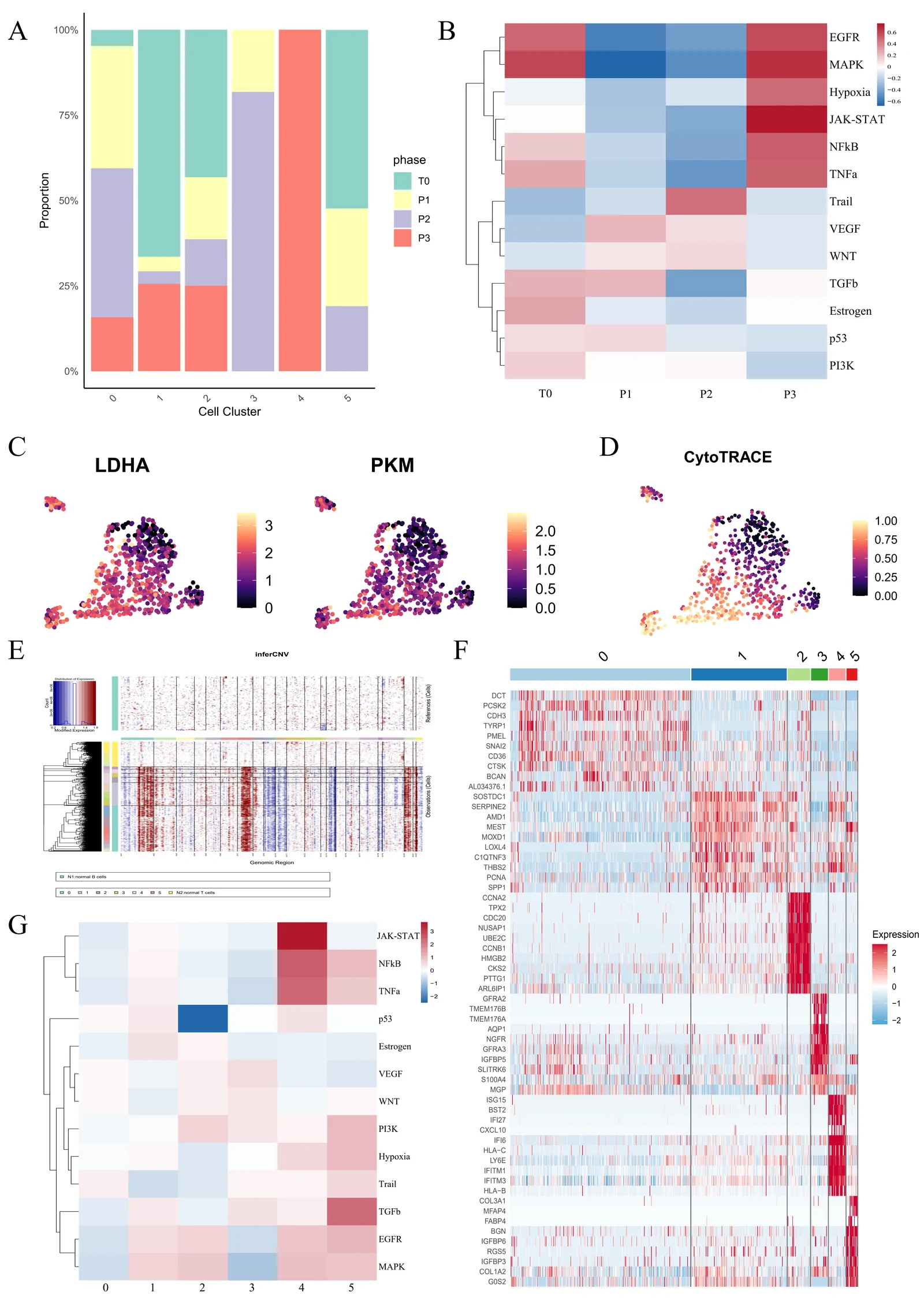
Functional characterization of treatment-associated melanoma cell states. (**A**) Proportional distribution of Clusters 0–5 across T0 (untreated), P1 (regression), P2 (minimal residual disease), and P3 (resistant regrowth). (**B**) PROGENy-inferred activities of 14 signaling pathways across the four treatment phases. (**C**) UMAP visualization of *LDHA* and *PKM* expression. (**D**) CytoTRACE scores projected onto the UMAP; higher scores indicate a predicted less differentiated transcriptional state. Cluster-wise score distributions are shown in Figure S3. (**E**) inferCNV profiles across the six clusters, with normal B cells (N1) and T cells (N2) used as references. (**F**) Heatmap of the top cluster-specific differentially expressed genes (Wilcoxon rank-sum test, Bonferroni-adjusted *p* < 0.05). (**G**) PROGENy-inferred pathway activities across Clusters 0–5.

To further define the melanoma cell states associated with therapeutic response and resistance, the distribution of the six clusters across the four treatment phases was examined (Figure 2A and Table S2). Clusters 0 and 3 were predominantly enriched during the initial regression and MRD phases (P1 and P2), consistent with treatment-responsive cell states. Cluster 1 was present across all four phases but was enriched at T0 and P3, suggesting a pre-existing state retained during treatment. Cluster 2 was also detected across all four phases, indicating a potential baseline state rather than a purely therapy-associated population. All 33 Cluster 4 cells were detected at P3 and represented 22.3% of the P3 population; the remaining P3 cells were distributed across Clusters 0, 1, and 2, indicating substantial heterogeneity at resistant regrowth (Table S2). Based on its exclusive detection at P3, Cluster 4 was provisionally considered a candidate acquired resistance-associated state that might differ from the other P3 populations and was selected for subsequent multidimensional characterization.

Pathway activities were next inferred across treatment phases to characterize signaling changes associated with resistant regrowth (Figure 2B). PROGENy-inferred MAPK pathway activity decreased during the regression phases and increased again at P3, consistent with pathway reactivation during resistant regrowth in the MEL006 model. JAK-STAT and hypoxia-related pathways, which showed relatively low inferred activity before treatment, also showed increased inferred activity at P3. Other inflammatory and stress-related pathways, including NF-κB and TNF-α signaling, showed sustained inferred activity at P3. These findings indicated that resistant regrowth was accompanied by coordinated pathway reprogramming rather than simple restoration of the untreated state.

To identify the cellular populations contributing to these resistant phenotypes, metabolism, differentiation potential, and inferred copy-number patterns were further characterized across clusters. Elevated expression of glycolytic markers, including *LDHA* and *PKM*, was observed in Clusters 1, 2, and 4 (Figure 2C), together with higher metabolic pathway scores (Figure S4), consistent with increased metabolic activity in these populations. CytoTRACE scores differed across the six clusters (Kruskal–Wallis *p* < 0.0001; Figure S3). Compared with Clusters 0, 3, and 5, Clusters 1, 2, and 4 collectively showed higher scores (two-sided Wilcoxon rank-sum test, *p* < 2.2 × 10^−16^; Figure 2D and Figure S3), consistent with a relatively less differentiated, stem-like transcriptional state. In contrast, inferCNV analysis did not reveal marked cluster-specific differences in large-scale CNV patterns (Figure 2E), indicating that the pronounced transcriptional heterogeneity was not accompanied by similarly distinct copy-number profiles.

Distinct transcriptional programs further separated the resistance-associated clusters. Cluster 2 was characterized by increased expression of cell-cycle-related genes, such as *CCNB1* and *CDC20* (Figure 2F), together with reduced p53 pathway activity (Figure 2G), consistent with a proliferative state potentially associated with pre-existing resistance. By contrast, Cluster 4 showed strong induction of interferon-stimulated genes (ISGs), including *ISG15* and *IFI27* (Figure 2F), together with elevated inferred activities of the JAK-STAT, NF-κB, and TNF-α signaling pathways (Figure 2G). Taken together, its exclusive detection at P3, the MAPK reactivation observed during resistant regrowth, and its interferon-associated transcriptional and pathway profile supported the interpretation of Cluster 4 as a candidate therapy-associated acquired resistance state. These features prompted further investigation of the transcriptional regulators associated with this state.

### 2.3. Interferon-Associated Regulatory Programs and Predicted Links to MAPK Pathway Reactivation

**Figure 3:**
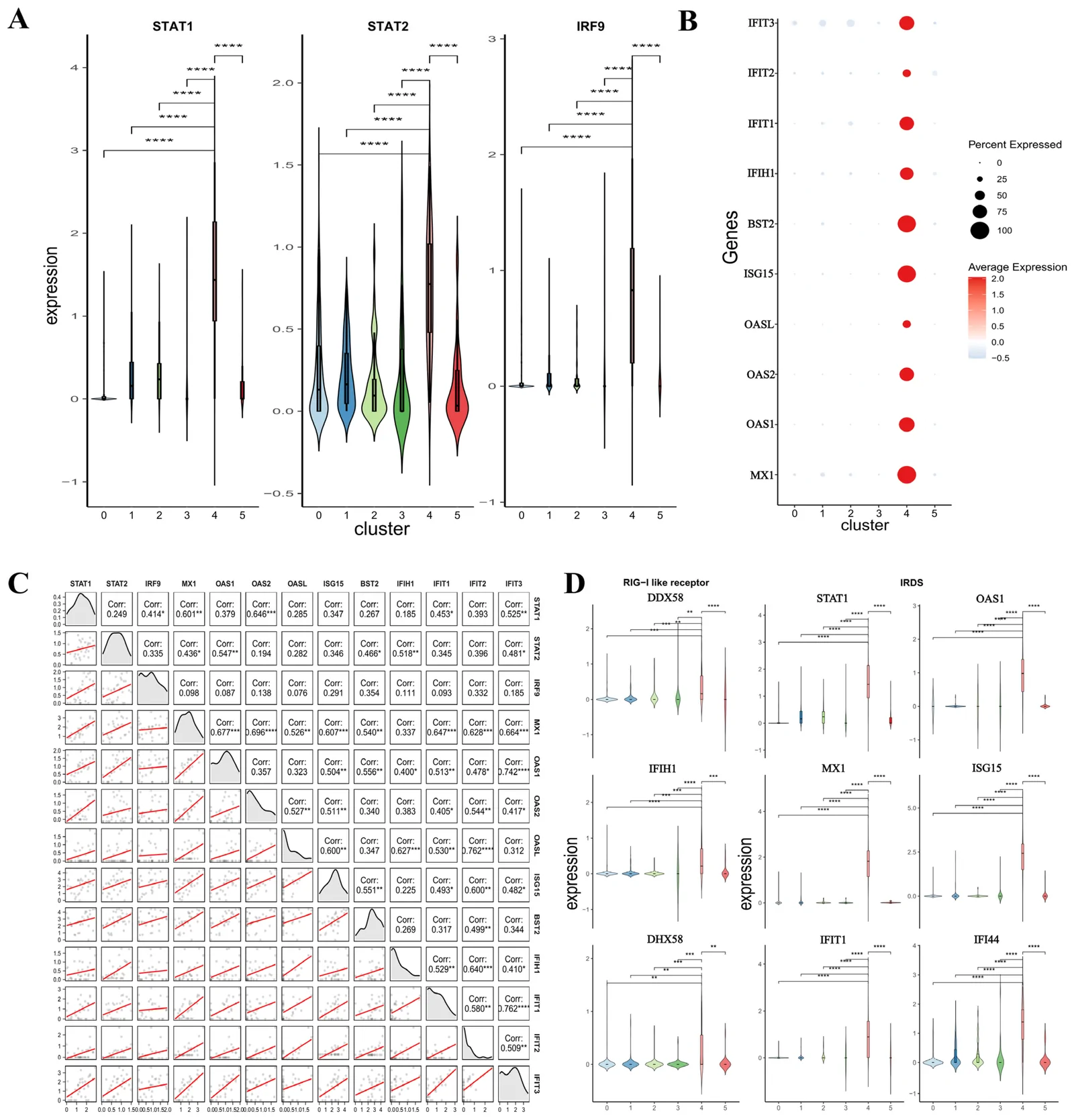
Interferon-associated transcriptional features of Cluster 4. (**A**) Expression of *STAT1*, *STAT2*, and *IRF9* across Clusters 0–5. (**B**) Expression frequency and average expression of representative interferon-stimulated genes. (**C**) Pairwise Pearson correlations among ISGF3 components and downstream ISGs in Cluster 4. (**D**) Expression of RIG-I-like receptor and IRDS-associated genes across clusters. In (**A**) and (**D**), Cluster 4 was compared with each other cluster using two-sided Welch’s *t*-tests, followed by Benjamini–Hochberg correction across 15 and 45 comparisons, respectively. Correlation *p* values in (**C**) were corrected jointly with those in Figure S6 (156 tests). Asterisks denote BH-adjusted *p* values: * *p* < 0.05, ** *p* < 0.01, *** *p* < 0.001, and **** *p* < 0.0001.

To characterize the regulatory program associated with Cluster 4, the expression of interferon-related transcription factors and their downstream genes was examined. *STAT1*, *STAT2*, and *IRF9* were identified as Cluster 4 marker genes and mapped to the canonical KEGG JAK-STAT signaling pathway (Figure S5). Their expression was higher in Cluster 4 than in the other clusters (Figure 3A). These transcription factors constitute the ISGF3 complex, which regulates interferon-stimulated gene expression [19,20]. Consistently, canonical ISGs, including *MX1* and *ISG15*, were highly expressed in Cluster 4 (Figure 3B). After Benjamini–Hochberg correction across the correlation tests in Figure 3C and Figure S6, several positive correlations between ISGF3 components and downstream ISGs remained significant in Cluster 4, whereas fewer were retained in non-Cluster 4 cells. These findings supported a coordinated interferon-associated transcriptional program in Cluster 4.

Cluster 4 also showed increased expression of RIG-I-like receptor genes and six representative IRDS-associated genes (Figure 3D) [21,22]. After joint Benjamini–Hochberg correction across Figure S7A,B, several positive correlations remained significant in Cluster 4, whereas the corresponding correlation coefficients were generally smaller in non-Cluster 4 cells. These results provided additional evidence of *STAT1*-associated interferon signaling in this state.

**Figure 4:**
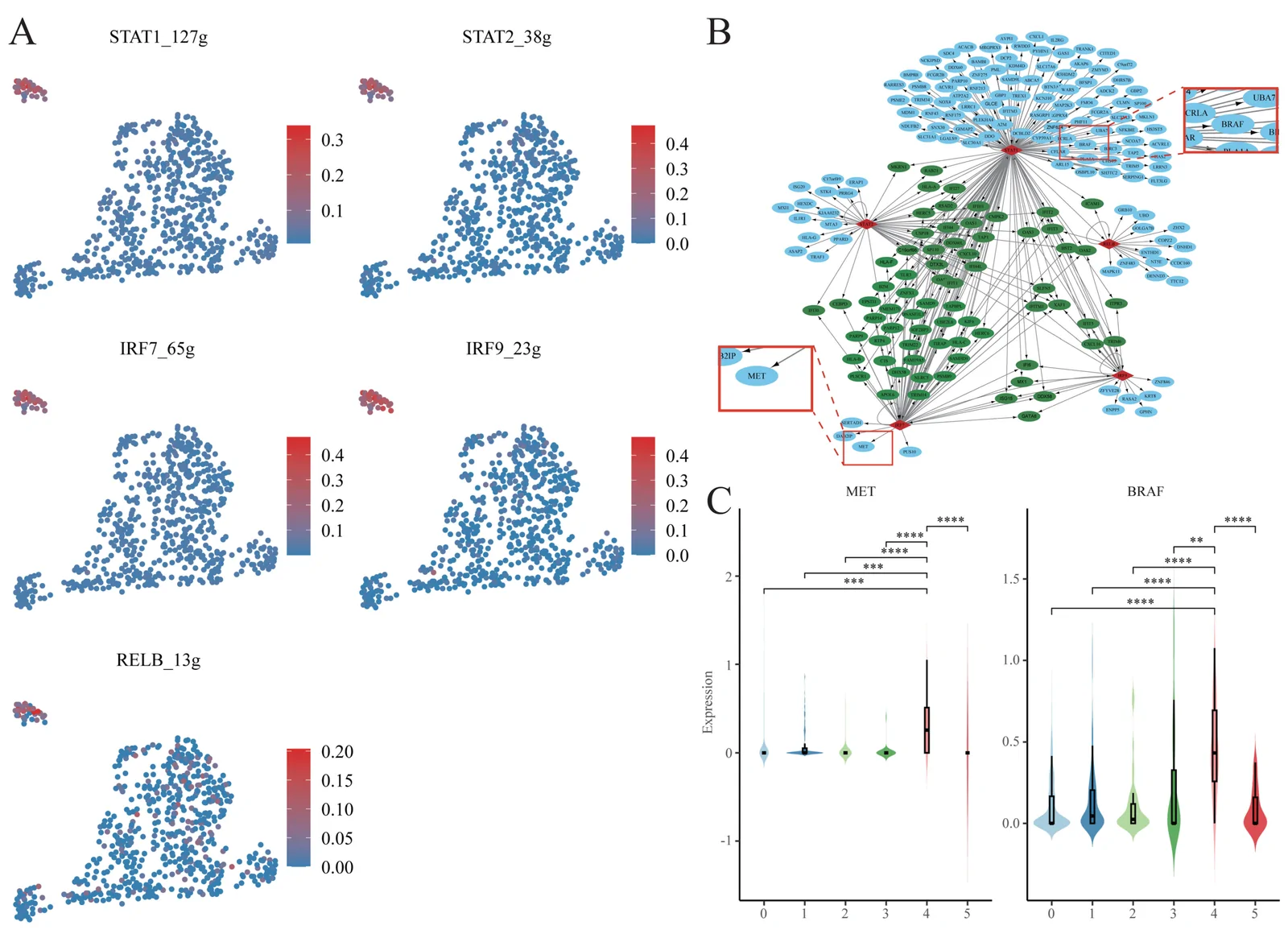
SCENIC-inferred regulons and candidate regulatory targets in Cluster 4. (**A**) UMAP projections of AUCell scores for the *STAT1*, *STAT2*, *IRF7*, *IRF9*, and *RELB* regulons. The numbers following the underscores in the regulon labels indicate the numbers of predicted target genes. (**B**) Predicted transcription factor–target network. Transcription factors, individual targets, and shared targets are shown as red diamonds, blue ovals, and green ovals, respectively. Dashed insets highlight the predicted *STAT1*-*BRAF* and *IRF7*-*MET* links. (**C**) Expression of the candidate regulatory targets *BRAF* and *MET* across Clusters 0–5. Cluster 4 was compared with each other cluster using two-sided Welch’s *t*-tests, followed by Benjamini–Hochberg correction across 10 comparisons. Asterisks denote BH-adjusted *p* values: * *p* < 0.05, ** *p* < 0.01, *** *p* < 0.001, and **** *p* < 0.0001.

SCENIC analysis identified higher inferred activities of the *STAT1*, *STAT2*, *IRF7*, *IRF9*, and *RELB* regulons in Cluster 4 (Figure 4A). This regulon pattern was consistent with the interferon-associated and NF-κB pathway activities identified above. A transcription factor–target network constructed from the SCENIC predictions linked *STAT1* to *BRAF* and *IRF7* to *MET*, in addition to multiple canonical ISG targets (Figure 4B). Both *BRAF* and *MET* were expressed at significantly higher levels in Cluster 4 than in the other clusters after Benjamini–Hochberg correction (Figure 4C). Together, these predicted links and expression differences nominated *STAT1*-*BRAF* and *IRF7*-*MET* as candidate regulatory relationships that could connect the Cluster 4 program to MAPK pathway reactivation through increased *BRAF* expression or *MET*-mediated bypass signaling [23].

Because no public dataset directly tested the complete proposed regulatory and drug-response model, four partially matched perturbation resources were reanalyzed (Figure S8 and Table S3). Across the *STAT1* perturbation datasets, evidence for a corresponding reduction in *BRAF* expression was limited. In SCP1064, *BRAF* expression showed a nonsignificant decrease after *STAT1* targeting (0.580 versus 0.806 ln (CPM + 1), *p* = 0.296), although negative effect estimates were obtained in 96 of 100 matched-control downsamplings and for all three *STAT1*-targeting sgRNAs (Figure S8A–D). The single *STAT1*-siRNA sample in GSE31534 showed no corresponding changes in *BRAF*, *MET*, or inferred MAPK pathway activity. Goh et al.’s CRISPR screen also showed nominal enrichment of *STAT1*-targeting guides during vemurafenib treatment (LFC = 0.766, *p* = 0.040), but this effect did not remain significant after multiple-testing correction (FDR = 0.617). The predicted *IRF7*-*MET* relationship could not be evaluated because effective *IRF7* suppression was not observed in GSE151825. Overall, these analyses provided directional but inconclusive support for the predicted *STAT1*-*BRAF* relationship, whereas no interpretable evidence was obtained for *IRF7*-*MET*.

### 2.4. Cross-Dataset Evaluation of the Cluster 4-Associated Transcriptional Program

**Figure 5:**
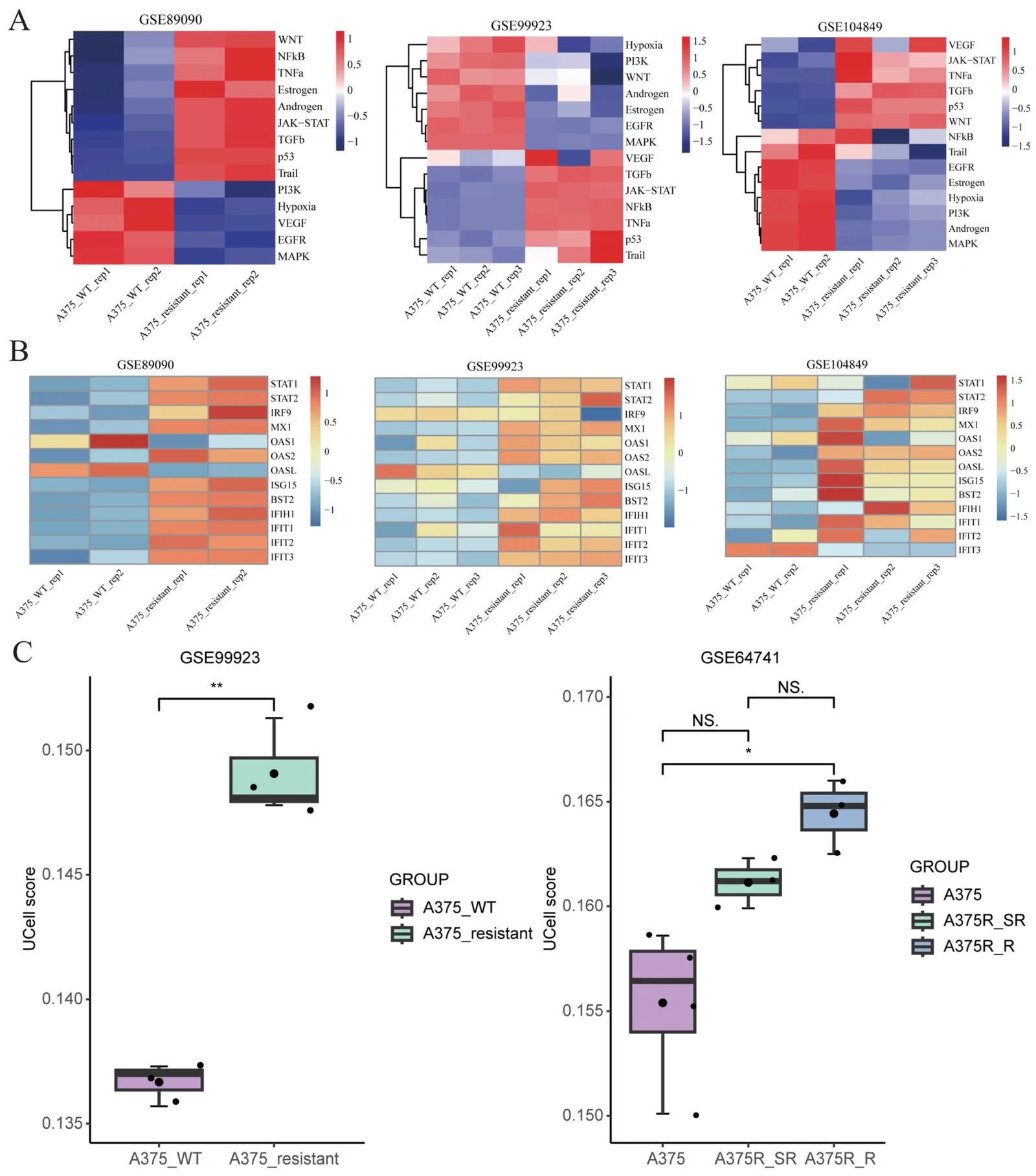
Cross-dataset evaluation of the Cluster 4-associated transcriptional program. (**A**) PROGENy-inferred pathway activities in sensitive and drug-treated or resistant states from GSE89090, GSE99923, and GSE104849. GSE89090 and GSE99923 represent stable acquired-resistance models, whereas GSE104849 represents a short-term adaptive response. The datasets were analyzed separately. PROGENy scores reflect transcriptional pathway output rather than direct MEK or ERK phosphorylation. (**B**) Expression of ISGF3 components and representative downstream ISGs. (**C**) UCell-based Cluster 4 signature scores in GSE99923 and the selected A375/A375R subsets of GSE64741. Boxes indicate medians and interquartile ranges. GSE99923 was analyzed using a two-sided Welch *t*-test. GSE64741 was analyzed using an omnibus one-way ANOVA followed by three prespecified pairwise Welch’s *t*-tests. Reported pairwise *p* values were not adjusted: * *p* < 0.05, ** *p* < 0.01; NS, not significant.

To assess whether the interferon-associated component of the Cluster 4 program was detectable in independent models, transcriptomic datasets from *BRAF* V600E-mutant melanoma cell lines representing stable acquired resistance or short-term drug exposure were analyzed. In several comparisons, drug-treated, drug-tolerant, or resistant populations showed higher inferred JAK-STAT, NF-κB, and TNF-α pathway activities than the corresponding controls (Figure 5A). Increased expression of ISGF3 components, including *STAT1*, *STAT2*, and *IRF9*, and canonical ISGs, such as *MX1* and *ISG15*, was also observed in several datasets (Figure 5B). By contrast, inferred MAPK pathway activity varied across models and was reduced in several comparisons (Figure 5A). Thus, the interferon-associated component recurred in several external models, whereas MAPK reactivation was not uniformly reproduced.

To further quantify the transcriptional similarity between external resistant models and Cluster 4, a Cluster 4 signature score was calculated using the UCell method. Resistant or drug-tolerant A375-derived populations showed higher Cluster 4 signature scores than their treatment-sensitive counterparts (Figure 5C). These findings further supported the partial reproducibility of the Cluster 4-associated transcriptional program across independent melanoma models.

### 2.5. A Pre-Existing Proliferative Resistance-Associated State Is Associated with Shorter Progression-Free Survival

**Figure 6:**
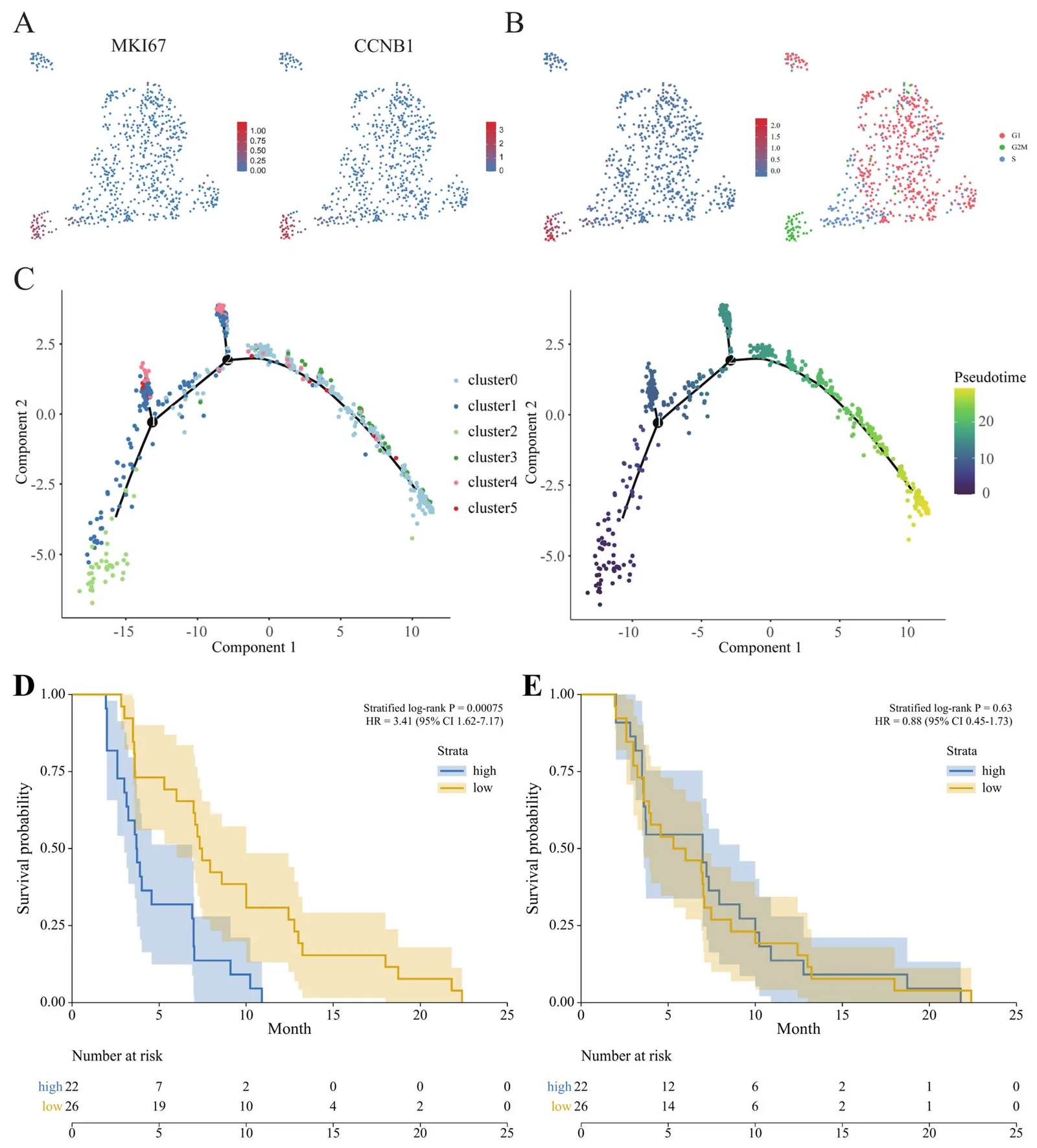
Proliferative features, pseudotime organization, and clinical relevance of resistance-associated melanoma cell states. (**A**) UMAP visualization of *MKI67* and *CCNB1* expression. (**B**) Cell-cycle states projected onto the UMAP, shown as relative score density (left) and discrete G1, S, and G2/M assignments (right). (**C**) Monocle2-inferred pseudotime organization of melanoma cells, colored by cluster identity (left) or pseudotime (right). The inferred topology represents transcriptional-state relationships rather than experimentally traced lineage transitions. (**D**,**E**) Kaplan–Meier PFS curves and number-at-risk tables for the Cluster 2 and Cluster 4 signatures in 48 pretreatment *BRAF* V600E-mutant melanoma samples. High- and low-score groups were defined within each GEO cohort, and survival analyses were stratified by cohort. (**D**) A high Cluster 2 signature score was associated with shorter PFS (stratified log-rank *p* = 0.00075; HR for high versus low = 3.41, 95% CI 1.62–7.17). (**E**) The Cluster 4 signature was not significantly associated with PFS (stratified log-rank *p* = 0.63; HR = 0.88, 95% CI 0.45–1.73).

In addition to the P3-restricted, therapy-associated state represented by Cluster 4, Cluster 2 was detected at T0 and persisted across subsequent phases, consistent with a pre-existing state. Cluster 2 showed elevated expression of the proliferation markers *MKI67* and *CCNB1* and higher scores for established cell-cycle gene sets [24] (Figure 6A). Cell-cycle assignment further showed that Cluster 2 contained a greater proportion of G2/M-phase cells, whereas cells in the other clusters were more broadly distributed across G1 and S phases (Figure 6B). These observations characterized Cluster 2 as a melanoma cell population with a prominent proliferative transcriptional program.

Monocle2 pseudotime ordering positioned Clusters 2 and 4 on distinct branches, each connected to a subset of Cluster 1 (Figure 6C). This topology described inferred transcriptional-state relationships across therapeutic phases but did not establish Cluster 1 as a common progenitor or demonstrate direct conversion from Cluster 1 to either resistance-associated state. Their temporal classification was instead based on the observed phase distributions: Cluster 2 was detected at T0 and persisted across subsequent phases, whereas Cluster 4 was detected only at P3.

The clinical associations of these states were evaluated using pretreatment bulk transcriptomic data from 48 patients with *BRAF* V600E-mutant melanoma. After defining high- and low-score groups within each cohort and stratifying the survival analysis by cohort, patients with high Cluster 2 signature scores showed shorter PFS than those with low scores (stratified log-rank *p* = 0.00075; HR = 3.41, 95% CI 1.62–7.17; Figure 6D). In contrast, the Cluster 4 signature was not significantly associated with PFS in pretreatment samples (stratified log-rank *p* = 0.63; HR = 0.88, 95% CI 0.45–1.73; Figure 6E).

Together, the presence of Cluster 2 at T0 and the association of its signature with shorter PFS supported its interpretation as a pre-existing proliferative resistance-associated state. The absence of a corresponding association for the Cluster 4 signature in pretreatment samples was consistent with its P3-restricted occurrence in the PDX model. These temporal labels describe observed phase distributions and do not imply mutually exclusive developmental lineages.

## 3. Discussion

This study identified two complementary resistance-associated melanoma cell states during *BRAF*-targeted therapy. Cluster 4 was detected only at resistant regrowth and exhibited an interferon-associated transcriptional program, whereas Cluster 2 was present before treatment and showed a proliferative program associated with shorter progression-free survival. The spatial separation of these states was retained in the whole-transcriptome analysis, although the *BRAF*-centered feature set provided finer resolution of the cellular heterogeneity. Thus, the focused clustering strategy did not create the distinction between Clusters 2 and 4 but facilitated its identification within broader transcriptomic communities. Together, these findings support a model in which resistance involves both baseline cellular heterogeneity and therapy-associated transcriptional reprogramming.

The transcriptional profile of Cluster 4 suggested that interferon-associated signaling was an important component of the therapy-associated state. Increased expression of ISGF3 components and downstream ISGs was accompanied by RIG-I-like receptor and IRDS-associated genes, together with higher inferred activities of the *STAT1*, *STAT2*, *IRF7*, and *IRF9* regulons. These features are consistent with previous evidence that stress-induced interferon signaling can contribute to therapeutic adaptation [21,22]. Concurrent *RELB* regulon activity and inferred NF-κB and TNF-α pathway activities further indicate that the Cluster 4 program extended beyond canonical ISGF3 signaling to include broader inflammatory transcriptional reprogramming. Related interferon-response and TNF-α/NF-κB programs have been reported in melanoma with poor response or resistance to *BRAF*/MEK-targeted therapy [25]. The partial recurrence of these features in independent cell-line models supports their relevance across several resistance contexts.

MAPK pathway reactivation, by contrast, was not uniformly reproduced in the external datasets. This difference does not necessarily contradict the MEL006 findings because the external models varied in treatment duration and reported resistance mechanism, ranging from short-term adaptive responses to stable resistant derivatives. PROGENy also estimates transcriptional pathway output rather than direct MEK or ERK phosphorylation. MAPK reactivation should therefore be interpreted as a feature of resistant regrowth in the MEL006 model, whereas the interferon-associated component appears to be the more reproducible aspect of the Cluster 4 program across the evaluated models.

Within this program, the predicted *STAT1*-*BRAF* and *IRF7*-*MET* links provide possible connections to MAPK pathway reactivation. Increased *BRAF* expression could reinforce pathway signaling, whereas *MET* may provide a receptor tyrosine kinase-mediated bypass route [23]. However, these relationships were inferred from regulon and transcription factor–target networks and remain distinct from direct transcriptional regulation. The public perturbation resources provided only directional and inconclusive support for the predicted *STAT1*-*BRAF* relationship, while ineffective *IRF7* suppression prevented meaningful evaluation of *IRF7*-*MET*. These observations refine the proposed mechanism but do not establish causality. The two links should therefore be regarded as candidates for functional validation rather than confirmed components of acquired resistance.

Cluster 2 represented a different aspect of resistance-associated heterogeneity. Its presence at T0, persistence across subsequent phases, proliferative transcriptional profile, and association with shorter PFS support its interpretation as a pre-existing proliferative resistance-associated state. In contrast, the Cluster 4 signature was not associated with PFS in pretreatment samples, consistent with its restriction to resistant regrowth in the PDX model. The pseudotime topology placed Clusters 2 and 4 on distinct branches connected to subsets of Cluster 1, but this organization describes transcriptional-state relationships rather than experimentally traced lineage transitions. It neither establishes Cluster 1 as a common progenitor nor demonstrates direct conversion between the states. Likewise, the higher CytoTRACE scores in Clusters 1, 2, and 4 and the absence of marked cluster-specific inferCNV differences are compatible with transcriptional plasticity but do not define its direction or underlying mechanism. Accordingly, “pre-existing” and “therapy-associated” describe temporal occurrence rather than mutually exclusive developmental lineages.

Several limitations constrain these interpretations. The discovery dataset was derived from six mice carrying a single patient-derived MEL006 PDX lineage. Animal allocation by phase and cell-level animal identifiers were unavailable, precluding replicate-aware pseudobulk or mixed-effects analyses. Cell-level *p* values should therefore be interpreted descriptively rather than as animal-level inference. Missing plate and sequencing-batch identifiers also prevented formal evaluation of phase-batch confounding, and estimates from clusters containing only 21–33 cells may be less stable. Replicate-aware analyses across additional independently derived PDX models will be required to establish the reproducibility of the identified states.

The proposed regulatory mechanism also lacks direct functional validation. No available dataset simultaneously included validated *STAT1* or *IRF7* perturbation, measurement of *BRAF* and *MET* expression, assessment of MAPK activity, and BRAFi/MEKi response in a *BRAF*-mutant melanoma model. Perturbation experiments combined with drug-response assays and direct measurements of MAPK signaling are required to determine functional relevance. ChIP-qPCR, CUT&RUN, or CUT&Tag experiments will also be needed to establish whether *STAT1* and *IRF7* occupy regulatory regions associated with *BRAF* and *MET*.

Finally, biological and clinical generalizability remains limited by differences among PDX tumors, cultured cell lines, and bulk clinical cohorts. The tumor-intrinsic analysis did not capture immune or stromal contributions to resistance, while the external cell-line models represented heterogeneous treatment durations and resistance mechanisms. Although clinical expression profiles were collected before treatment, subsequent treatment could influence PFS. Most cohorts were homogeneous by treatment class, but GSE99898 included both BRAFi monotherapy and combined BRAFi/MEKi therapy, and its small treatment subgroups precluded reliable multivariable adjustment. Validation in larger longitudinal cohorts with standardized treatment and sampling will therefore be necessary.

## 4. Materials and Methods

### 4.1. Data Sources and Patient Cohorts

#### 4.1.1. Longitudinal Single-Cell Discovery Dataset

The primary single-cell RNA sequencing dataset was obtained from the Gene Expression Omnibus (GEO) repository under accession number GSE116237 [16]. This dataset comprises high-resolution transcriptomes of 674 melanoma cells derived from a longitudinal patient-derived xenograft (PDX) model carrying the *BRAF* V600E mutation. Cells were isolated across four therapeutic checkpoints during BRAFi/MEKi treatment: untreated (T0), initial regression (P1), minimal residual disease (P2), and resistant regrowth (P3). The original study generated ten 96-well plates from six PDX-bearing mice and sequenced them in three batches. Because the public metadata do not retain cell- or plate-level animal identifiers or definitive animal-to-phase assignments, phase-specific biological replication could not be reconstructed.

#### 4.1.2. External Cell-Line Comparison Datasets

Four independent transcriptomic datasets containing A375-derived melanoma models were obtained from GEO for cross-dataset validation of the resistance-associated transcriptional program. A375 is a widely used *BRAF* V600E-mutant melanoma cell line. GSE89090 compared parental A375 cells with A375DTR cells exhibiting acquired resistance to combined dabrafenib and trametinib [26], whereas DMSO-treated controls and short-term vemurafenib-treated A375 samples, representing an adaptive response rather than stable resistance, were selected from GSE104849 [27]. GSE99923 compared parental A375 cells with PLX4720-resistant clonal populations [28], whereas relevant A375/A375R samples were selected from GSE64741 [29]. Because these datasets differed in profiling platform, treatment regimen, exposure duration, resistance state, and reported mechanism, each was analyzed separately. They were used to assess partial recurrence of the Cluster 4 program rather than uniform MAPK reactivation.

#### 4.1.3. Independent Perturbation and CRISPR-Screen Datasets

Because no public dataset jointly included a *BRAF*-mutant melanoma model, validated *STAT1* or *IRF7* perturbation, *BRAF*/*MET* expression, MAPK activity, and BRAFi/MEKi response, four partially matched resources were analyzed. SCP1064 contained 110 untreated cells carrying three *STAT1*-targeting sgRNAs and matched non-targeting controls from patient-derived *BRAF* V600E melanoma model 2686 [30]. GSE31534 contained one *STAT1*-siRNA and three control A375 samples collected 48 h after transfection [31]. GSE151825 contained three *IRF7*-targeted and ten control WM989 samples [32]. Goh et al.’s screen contained A375 cells treated with vemurafenib or DMSO for 7 or 14 days, with two replicates per condition and time point [33].

#### 4.1.4. Pretreatment Clinical Validation Cohort

For clinical translation and survival analysis, public clinical transcriptomic and survival data from 48 *BRAF* V600E-mutant melanoma patients were collected across six independent cohorts: GSE50509 [34], GSE50535 [6], GSE61992 [35], GSE65185 [36], GSE77940 [37], and GSE99898 [38] (Table S4). All expression data represented baseline, pretreatment tumor biopsies and were integrated with matched progression-free survival (PFS) records. Treatment was homogeneous at the monotherapy-versus-combination level within each cohort except GSE99898. Although treatment could not affect pretreatment signature scores, it could influence subsequent PFS.

### 4.2. Computational and Bioinformatic Analyses

#### 4.2.1. BRAF-Centered Network Feature Set Construction

To investigate cell-state characteristics under *BRAF* inhibition, a *BRAF*-centered candidate network was constructed to guide unsupervised clustering. The human *BRAF* protein identifier (ENSP00000288602) was queried against STRING v11.5 [39] with a combined interaction score threshold of ≥900. First- and second-order physical and functional partners were mapped to standard human gene symbols (Table S5). Intersecting this network with genes represented in the primary scRNA-seq matrix yielded 2506 candidate genes. Coverage was evaluated against the Reactome RAF activation (R-HSA-5673000) and RAF/MAP kinase cascade (R-HSA-5673001) gene sets after HGNC symbol normalization.

#### 4.2.2. Single-Cell Preprocessing, Clustering, and Marker Identification

The raw single-cell expression count matrix was processed in R using the Seurat package [40]. Gene expression was normalized using LogNormalize with a scale factor of 10,000. For cell clustering, variable-feature selection was restricted to the 2506-gene *BRAF*-centered candidate set, from which the top 1000 highly variable genes were selected. The first 16 principal components were used to construct a shared nearest-neighbor graph, and Louvain clustering was performed at a resolution of 0.31. UMAP was used to visualize the resulting six clusters. Cluster-specific marker identification and all subsequent analyses used the full-transcriptome expression matrix.

Resolution sensitivity was evaluated from 0.20 to 0.40 in 0.01 increments using clustree with otherwise unchanged parameters. The six-cluster structure was stable at resolutions of 0.28–0.31, with additional fragmentation from 0.32. A whole-transcriptome sensitivity analysis used the top 1000 unrestricted highly variable genes with the same PCA, resolution, and UMAP settings; the *BRAF*-centered cluster labels were projected onto this UMAP for comparison.

Cluster-specific marker genes were identified using a minimum detection fraction (min.pct) of 0.25, a log-fold-change threshold (logfc.threshold) of 0.25, and the Wilcoxon rank-sum test. Genes with a Bonferroni-adjusted *p* value < 0.05, as reported by Seurat, were retained.

Because public metadata lacked cell-level animal identifiers, animal-level pseudobulk or mixed-effects analyses could not be performed reliably. Cell-level Wilcoxon tests, Welch’s *t*-tests, and correlations were therefore interpreted descriptively; multiple-testing correction does not substitute for biological replication.

#### 4.2.3. Pathway Activity, Metabolic State, and IRDS-Associated Expression Analysis

Pathway activities across treatment phases and clusters were inferred using PROGENy [41] with the human model and top 500 responsive genes per pathway. Metabolic pathway scores were computed using curated KEGG gene sets [42], and *LDHA* and *PKM* expression was evaluated separately. Cluster 4 marker genes were assigned a binary value of 1 and mapped onto KEGG hsa04630 using Pathview [43]; highlighted nodes indicate marker membership rather than expression magnitude or statistical significance.

Scaled full-transcriptome expression was visualized for six genes from the focused seven-gene IRDS panel reported by Boelens et al. [21] (*STAT1*, *OAS1*, *MX1*, *ISG15*, *IFIT1*, and *IFI44*). *IFIT3* is shown in Figure 3B and included in Figure 3C. No score based on the complete 49-gene IRDS [44] was calculated.

Cluster 4 was compared with each other cluster using two-sided Welch’s *t*-tests. Benjamini–Hochberg correction was applied within Figure 3A (15 comparisons), Figure 3D (45), and Figure 4C (10). Pearson correlation *p* values were corrected jointly across Figure 3C and Figure S6 (156 tests) and across Figure S7A and Figure S7B (72 tests). Figure 5C used one Welch t-test for GSE99923 and three prespecified Welch tests after omnibus ANOVA for GSE64741, without an additional across-dataset correction.

#### 4.2.4. Differentiation Potential and Copy-Number Variation Analysis

The relative differentiation potential of individual melanoma cells was estimated using CytoTRACE [45], with higher scores indicating a predicted less differentiated state. Overall cluster differences were evaluated using the Kruskal–Wallis test, and Clusters 1, 2, and 4 were compared collectively with Clusters 0, 3, and 5 using a two-sided Wilcoxon rank-sum test. Large-scale copy-number variations were inferred using inferCNV, with normal B cells (N1) and T cells (N2) as references. A cutoff of 1, denoising, and hidden Markov model prediction were used.

#### 4.2.5. Transcription Factor Regulon Network Analysis

Transcription factor regulon activities were inferred using SCENIC [46]. Co-expression modules were generated using GENIE3 and filtered by RcisTarget with hg19 cisTarget databases covering 500 bp upstream and 10 kb around transcription start sites. AUCell quantified single-cell regulon activities. *STAT1*, *STAT2*, *IRF7*, *IRF9*, and *RELB* were prioritized based on enrichment in Cluster 4 and predicted regulatory links with resistance-associated genes, including *BRAF* and *MET*.

#### 4.2.6. Cluster 4 Signature Scoring and External Cross-Dataset Evaluation

A Cluster 4 signature score was calculated using UCell [47], which estimates rank-based gene-set enrichment within an expression matrix (Table S5). The score was projected onto the external bulk RNA-seq and microarray datasets to compare treatment-sensitive, drug-treated, and resistant cell-line models, as appropriate for each dataset.

#### 4.2.7. Orthogonal Perturbation and CRISPR-Screen Reanalysis

For SCP1064, 110 untreated *STAT1*-targeted cells were compared with 110 downsampled non-targeting controls using two-sided Mann–Whitney U tests on ln (CPM + 1) expression. The *BRAF*-expression difference was evaluated across 100 matched-control downsamplings and separately for each sgRNA; cell-level *p* values were treated as descriptive. GSE31534 RMA expression was log2 transformed and median-centered within expression-inferred batches before comparing one *STAT1*-siRNA sample with three controls. GSE151825 was analyzed using a negative-binomial model and preranked pathway analysis. The Goh screen was reanalyzed with MAGeCK; day 14 vemurafenib versus DMSO was the primary contrast.

#### 4.2.8. Pseudotime Trajectory and Cell-Cycle Analysis

Single cells were assigned to G1, S, or G2/M phases using Seurat cell-cycle scoring and canonical markers. Pseudotime analysis was conducted using Monocle2 [48] across all four therapeutic phases. Single-cell expression matrices were filtered to retain genes detected in more than 1% of the total cell population. Ordering genes were selected based on significant differential expression across the identified functional cell clusters to construct the linear or branching pseudotime trajectories.

#### 4.2.9. Pretreatment Clinical Survival and Prognostic Analysis

Cluster 2 and Cluster 4 signatures (Table S5) were scored by ssGSEA with GSVA [49]. The six cohorts were preprocessed and scored separately. GSE50509, GSE61992, and GSE99898 microarrays underwent cohort-specific offsetting, log2 transformation, quantile normalization, gene-symbol mapping, and median probe aggregation. GSE50535 and GSE65185 used log 2(TPM + 1), whereas GSE77940 RPKM values were rescaled to TPM and log2 transformed. Technical replicates were averaged. Within each cohort, patients were divided at the signature median; only group labels and clinical variables were pooled. Kaplan–Meier, log-rank, and Cox analyses were stratified by cohort, and proportional hazards were checked with Schoenfeld residuals. GSE99898 treatment effects were assessed descriptively because only six monotherapy and three combination-therapy patients were available.

## 5. Conclusions

In summary, two complementary resistance-associated melanoma cell states were identified: a pre-existing proliferative state associated with shorter progression-free survival and a therapy-associated acquired-resistance state characterized by interferon signaling. Within the MEL006 PDX model, the latter coincided with MAPK reactivation and yielded predicted *STAT1*-*BRAF* and *IRF7*-*MET* regulatory links. These links and their relationship to MAPK activity remain model-derived hypotheses that require functional and biochemical validation.

## Data Availability

All datasets analyzed in this study are publicly available in the Gene Expression Omnibus under accession numbers GSE116237, GSE89090, GSE104849, GSE99923, GSE64741, GSE50509, GSE50535, GSE61992, GSE65185, GSE77940, GSE99898, GSE31534, and GSE151825. The Perturb-CITE-seq dataset is available through the Broad Institute Single Cell Portal under accession SCP1064, and Goh et al.’s CRISPR-screen counts are available in the source publication’s supplementary files. No new datasets were generated. The *BRAF*-centered network and cluster-signature gene sets are provided in Supplementary Table S5.

## Supplementary Materials

The following supporting information can be downloaded at https://www.mdpi.com/article/10.3390/ijms27188031/s1.

## Abbreviations

BRAFi	BRAF inhibitor	MEKi	MEK inhibitor
PDX	patient-derived xenograft	MRD	minimal residual disease
scRNA-seq	single-cell RNA sequencing	MAPK	mitogen-activated protein kinase
CNV	copy-number variation	TF	transcription factor
ISG	interferon-stimulated gene	IRDS	interferon-related DNA damage resistance signature
PFS	progression-free survival	GEO	Gene Expression Omnibus
PROGENy	Pathway RespOnsive GENes	SCENIC	Single-Cell rEgulatory Network Inference and Clustering
AUCell	area under the curve-based gene-set activity scoring	UCell	Mann–Whitney U statistic-based single-cell signature scoring
GSVA	gene set variation analysis	ssGSEA	single-sample gene set enrichment analysis
RTK	receptor tyrosine kinase		
